# Can probiotics be used in the prevention and treatment of bronchial asthma?

**DOI:** 10.1007/s43440-024-00618-0

**Published:** 2024-07-01

**Authors:** Paulina Kleniewska, Rafał Pawliczak

**Affiliations:** Department of Immunopathology, Faculty of Medicine, Medical University of Lodz, Żeligowskiego 7/9, Łódź, 90-752 Poland

**Keywords:** Probiotics, Asthma, Dysbiosis, Microbiota

## Abstract

Asthma is a lifelong condition with varying degrees of severity and susceptibility to symptom control. Recent studies have examined the effects of individual genus, species, and strains of probiotic microorganisms on the course of asthma. The present review aims to provide an overview of current knowledge on the use of probiotic microorganisms, mainly bacteria of the genus *Lactobacillus* and *Bifidobacterium*, in asthma prevention and treatment. Recent data from clinical trials and mouse models of allergic asthma indicate that probiotics have therapeutic potential in this condition. Animal studies indicate that probiotic microorganisms demonstrate anti-inflammatory activity, attenuate airway hyperresponsiveness (AHR), and reduce airway mucus secretion. A randomized, double-blind, placebo-controlled human trials found that combining multi-strain probiotics with prebiotics yielded promising outcomes in the treatment of clinical manifestations of asthma. It appears that probiotic supplementation is safe and significantly reduces the frequency of asthma exacerbations, as well as improved forced expiratory volume and peak expiratory flow parameters, and greater attenuation of inflammation. Due to the small number of available clinical trials, and the use of a wide range of probiotic microorganisms and assessment methods, it is not possible to draw clear conclusions regarding the use of probiotics as asthma treatments.

## Introduction

According to the Global Burden of Disease Study [1], in 2019 asthma affected almost 270 million people, assuming an age-standardized prevalence of 3415,5 cases per 100,000 population. Despite the complexity of asthma etiology, many environmental factors associated with the development of the disease have been identified in recent years [2–5]. One factors involved in the etiopathology of asthma is the composition of intestinal microflora [6–8]. Clinical trials conducted in Sweden, the United Kingdom, and Japan indicated the presence of high levels of *Clostridium* and low levels of *Bifidobacterium* in infants who later developed allergies [9–11]. Additionally, experiments on animal models found that fermentation of dietary fiber by the *Bifidobacteriaceae* and *Lactobacillaceae* in the intestines increases the levels of short-chain fatty acids (SCFAs) [12, 13] which leads to a reduction in inflammation associated with the response of type 2 helper cells (Th2) [8, 14, 15]. SCFAs such as propionate, acetate and butyrate ameliorate allergic airway inflammation *via* regulatory T cells (Tregs) [16]. *Lactobacillus* and *Bifidobacterium* have been found to increase the secretion of interleukin (IL)-10 [17] and suppress the IgE-dependent immune response [18]. Probiotics are also known to affect natural killer (NK) cells, macrophages, monocytes, and T helper type 17 (Th17) cells; indeed, *L. paracasei* strains used in commercial products have been found to increase the expression of tumor necrosis factor-alpha (TNF-α), IL-6 or IL-8 in a human monocyte cell line, thus enabling an antimicrobial response [19]. It has also been shown that dendritic cells (DCs) cultured with *L. casei* and *L. reuteri* stimulate Tregs and Th1 [20]. *Lactobacillus* bacteria mainly act by stimulating DCs to secrete IL-12 [21, 22], while *Bifidobacterium* strains are more associated with the induction of an anti-inflammatory [23] and regulatory response *via* Tregs [24, 25]. In general, probiotics stimulate the secretion of interferon-gamma (INF-γ) [26] and IL-10 [27, 28], and support host immunity. *L. acidophilus* reduces IgE secretion [29–31]. In addition, *Lactobacillaceae* biosynthesize vitamins such as B12, B9, B7 and support the absorption of magnesium, calcium and, iron by the host [32, 33].

The present study reviews the latest data on the role of probiotics in asthma prevention and treatment and the impact of selected bacterial strains on bronchial asthma. The sources include various animal models and clinical trials involving children. It highlights the problems associated with the homogeneity of existing data, and proposes changes that can be implemented to best exploit the potential of probiotics in asthma therapy.

## Asthma– an overview

Asthma was the cause of 455,000 deaths (almost 1000 deaths per day in 2019), due to inappropriate asthma management, mostly in low- and middle-income countries [2, 34]. In 2017, the annual costs of asthma treatment in the USA were estimated at about 3,100 USD per patient [35].

The symptoms are related to chronic airway inflammation [36, 37] which is enhanced by numerous cytokines secreted by cells such as macrophages, eosinophils, neutrophils, and lymphocytes [38, 39]. These can damage the epithelium and other structures, and exacerbate bronchial tree remodeling [40–42]. Patients experience excessive bronchoconstriction in response to stimuli, manifested as episodes of shortness of breath accompanied by wheezing and coughing, especially at night and in the morning. An isolated cough may be the only symptom of the disease in children (cough-variant asthma) [43–45].

Asthma develops by a multifactorial process associated with chronic inflammation of the airway with extensive infiltration of eosinophils, mast cells, and T lymphocytes. This limits airflow associated with smooth muscle contraction, mucosal swelling, excess mucous secretion, and airway remodeling, and leads to bronchial hyper-reactivity [46–50].

During asthma, the mucus layer thickens and the smooth muscle layer becomes overgrown. Additionally, prolonged inflammation causes subepithelial fibrosis of the bronchial tree, reducing its elasticity and preventing easy contraction and relaxation [51–53].

Classically, asthma is divided into allergic or non-allergic phenotypes; these differ in regard to age at onset, the factor causing exacerbations, family history, and the presence of IgE antibodies. Bronchial asthma can also be classified into three types based on its cellular profile: eosinophilic, neutrophilic, and mixed eosinophilic/neutrophilic [54–56]. However, many types of asthma do not correspond directly to this classification, and therefore, a better picture of the disease is provided by a fivefold classification [57] based on clinical phenotype: allergic, non-allergic, fixed airflow limitation, late-onset, and obesity-associated disease. In clinical practice, asthma is usually classified based on the degree of control of its symptoms, which also can be used to indicate therapeutic success [53, 58]. Furthermore, the inflammatory phenotype can be classified as type 2, i.e. associated with eosinophils and IL-4, IL-5, and IL-13, and as non-type 2, i.e. associated more strongly with neutrophils [59–62].

### Treatment options in asthma

For years, treatment focused mainly on alleviating the symptoms of the disease [63]. Existing drugs can be divided into controlling, bronchodilating, and supportive medicines [57] depending on the role they play. These are mainly medications taken chronically, aimed at permanently controlling the disease (inhaled glucocorticosteroids, long-acting beta-2-agonists, and antileukotriene). Other medications such as inhaled short-acting beta-2-agonists, anticholinergic drugs, and oral corticosteroids can be taken by a patient *on demand* to provide relief. Finally, monoclonal antibodies directed against human IgE immunoglobulin, IL-5, or the IL-5 and IL-4 receptor may also be used in addition to standard medication in severe cases or when symptoms persist despite other medications [64]. The selection of medicines and doses depends on the disease phenotype and age of the patient, and may be influenced by a delay in the initiation of treatment. Furthermore, the initial therapy is typically modified after implementation depending on the patient response [65].

### An imbalance in the microbiota in asthma

Various respiratory diseases, including bronchial asthma, may also be induced by disturbances within the lung microorganism subpopulation [66–68]. A 2015 study found an increased number of *Proteobacteria* in the lower respiratory tract to be associated with worse asthma control and the occurrence of exacerbations, and that this was accompanied by the stimulation of genes related to Th17 cells [69]. Interestingly, asthmatics often demonstrate lower numbers of *Lactobacillaceae* bacteria, which are important for the development of regulatory T cells [70], and an increase in *Haemophilus* and *Neisseria* [71]. Hence, it is possible that the appropriate use of probiotics [72–74] containing *Lactobacillaceae* may have a beneficial effect on the microbiome and thus on asthma control.

Deregulation of intestinal microflora in early life has been repeatedly linked to the development of asthma in adults [68, 75, 76]. Arrieta et al. [77] found one hundred newborns at risk of asthma to have significantly lower numbers of *Veillonella*, *Faecalibacterium*, *Lachnospira*, and *Rothia* bacteria. Another study [78] found increased amounts of *Lachnospira* and *Clostridium neonatale* to be associated with childhood asthma. Research also indicates that patients suffering from gastrointestinal diseases are more likely to suffer from respiratory diseases [79], and that some bacteria present in the respiratory and gastrointestinal tract of newborns promote the maturation of the immune system and protect against the development of asthma [80].

## Studies on animal models assessing the use of probiotics in asthma

### *Lactobacillus rhamnosus*

In 2019, Spacova et al. [81] evaluated the potential of probiotic *Lactobacillus rhamnosus* GG and GR-1 strains for the preventive treatment of allergic asthma. Innovative intranasal administration was performed eight times per day. The researchers found the *L. rhamnosus* GG strain to bind to respiratory cells more readily, and have greater potential to translocate throughout the body; this has both positive and negative effects, as although administration may support the immune system, the bacteria are free to explore the host. Interestingly, the findings suggest that the beneficial effects of probiotics may extend beyond to the modulation of antibody production. *L. rhamnosus* GG treatment reduced the concentration of Betv1-specific IgG1, and prophylactic administration significantly reduced lung eosinophilic infiltration compared to an untreated group. The levels of eosinophils also fell in BALF, but the probiotic did not appear to have any effect on macrophages or neutrophils. The LGG treatment also significantly reduced the levels of IL-5 and IL-13. Prophylactic use of probiotics a few days before exposure to the allergen yielded positive results in the form of lower AHR. The study confirmed that preventive intranasal administration of *L. rhamnosus* GG may have the potential to alleviate the symptoms of allergic asthma.

Intranasal probiotic application may also strengthen the epithelial barrier of the respiratory tract, thus preventing the development of hypersensitivity. Studies have also shown that the effectiveness of some *Lactobacillus* strains differs depending on the method of administration [82].

A later study [83] examined the effects of orally-administered probiotic *Lactobacillus rhamnosus* GG (LGG) and/or the prebiotic turmeric (TP) on alleviating allergic inflammation. The research was conducted on a mouse model of asthma induced by house dust mites (HDM). BALB/c mice were treated with probiotic and prebiotic compounds, either separately or together. It was found that the combination of pro- and prebiotic inhibits the development of airway hyperreactivity in response to methacholine, but also significantly reduces the level of eosinophils, IL-5, IL-13, and CCL17. Moreover, the number of CD4 + Th2 and CD4 + Th17 cells in splenocytes was reduced compared to the control group. An increased frequency of CD25^+^ Foxp3^+^ Treg was observed *vs*. asthma, but also an increased number of Tregs *vs*. the probiotic group. The combination of prebiotic and probiotic had a better effect on inhibiting Th2 cells. These findings confirm that combined LGG + TP therapy is more effective in such conditions.

In 2022, Voo et al. [84] examined the effect of combined administration of *L. rhamnosus* GG with prednisolone, a synthetic glucocorticosteroid, on the course of allergic asthma in a Der p 2-sensitized asthma mouse model (female, BALB/c). The results indicated that the lower dose of prednisolone was less satisfactory in inhibiting AHR, serum IgE and IgG1 antibodies, Th2 cytokines, IL-6, IL-8, and IL-17, and infiltrating inflammatory cells. Combination therapy with LGG reduced airway resistance and IgE and IgG1 levels, and increased serum IgG2a levels; it also inhibited the production of IL-4, IL-5, IL-6, IL-8, IL-13, IL-17 and enhanced the Th1 immune response. The data indicates that LGG administration can reduce the dose of prednisolone and can be used in combination therapy in the course of asthma.

In 2023, Hou et al. [85] described the effect of the probiotic strain *Lactobacillus rhamnosus* 76 (LR76) in the course of asthma, particularly its involvement in the mechanism influencing mucus secretion. Experiments were performed on female BALB/c mice. Intragastric administration of the probiotic resulted in significantly reduced allergic inflammation of the respiratory tract and lower infiltration of inflammatory cells in lung tissue. The total cell count and percentage of eosinophils in BALF also decreased, as did mucus secretion in the respiratory tract and the expression of IL-4, IL-5, IL-13, IL-25, and TNF-α in the asthmatic mice. Treatment also reduced the expression of transforming growth factor-β1 (TGF-β1), IFN-γ, and IL-10 in the lung tissue. Importantly, the expression of STAT6/p-STAT6/SPDEF proteins and STAT6/SPDEF mRNA was significantly increased in IL-13-induced 16HBE cells and decreased after incubation with the probiotic.​

Another study [86] used a combination of a bespoke recombinant *Lactobacillus rhamnosus* GR-1 strain producing the major birch pollen allergen Bet v 1 together with a wild-type probiotic. Administration of both strains prevented the development of AHR. The recombinant strain also prevented increases in total airway cells count, lymphocytes, and lung IL-1β concentrations. The wild type inhibited airway eosinophilia. The recombinant probiotic did not alter the composition of the intestinal microbiome and had a modulating effect on asthma. The change in the composition of the intestinal microbiome correlated with the severity of inflammation and AHR. The findings indicate that the modified strain can prevent the deterioration of respiratory function in the course of the disease. The expression of Bet v 1 contributed to lower airway inflammation by reducing T helper 2-related responses. The authors suggest the involvement of additional mechanisms, and propose that the data suggests the existence of a lung-gut axis.

### *Lactobacillus paracasei*

In 2022, Chen et al. [87] examined whether *Lactobacillus paracasei* K47, now called *Lacticaseibacillus*, has an immunomodulatory effect in asthma. The heat-inactivated probiotic was administered orally to BALB/c mice. The authors demonstrated that the K47 strain significantly reduces serum concentrations of total IgE, OVA-specific IgE and OVA-specific IgG1 in asthmatic mice. Moreover, the strain inhibited the accumulation of inflammatory cells in BALF and alleviated AHR.

Another study [88] assessed the effect of orally- or intratracheally-administered *Lactobacillus paracasei* 33 on the course of disease. Rats with OVA-induced asthma received the probiotic for four weeks. Importantly, the animals were administered diesel exhaust particles intratracheally. Treatment resulted in a decrease in the number of inflammatory cells, lymphocytes and eosinophils in BALF, and a decrease in IgE concentration and cytokine levels in Th2 cells. No significant difference was noted in Th1 cell cytokine levels. The strain hence appears to have a significant impact on improving the symptoms of allergic asthma.

### *Lactobacillus plantarum*

In 2022, Lan et al. [89] evaluated the anti-asthmatic and anti-inflammatory effects of various doses of probiotic *Lactobacillus plantarum* CQPC11 on Balb/c mice. Treatment resulted in a reduction in AHR and lung W/D ratio in asthmatic mice. It also reduced the accumulation of inflammatory cells in BALF, attenuated histological edema, and reduced serum levels of OVA-specific IgE, IgE, and IgG1. Furthermore, probiotic treatment decreased the concentrations of TNF-α and IL-4, IL-5, IL-6 and IL-13 in BALF. The authors also report an increase in Foxp3 and T-bet mRNA levels and a decrease in Gata3 and RORγt mRNA levels in the lung. This was accompanied by a reduction in oxidative stress and a decrease in the activation of the NF-κB pathway.

A Japanese study [90] examined the influence of *Lactobacillus plantarum* RGU (now called *Lactiplantibacillus*) on the course of OVA-induced asthma. The experiment was performed on BALB/c mice. All animals except the control group received three broad-spectrum antibiotics for one week before intervention: penicillin (1000 U/ml), kanamycin (1 mg/ml), and streptomycin (1 mg/ml). Three probiotic strains were used in this experiment: *L. plantarum* RGU (Lp-1; isolated from naturally fermented milk), *L. plantarum* (Lp14917; as a control for the Lp-1 strain), and *Limosilactobacillus reuteri* (Lr; as the dominant species of intestinal bacteria from healthy mice). The probiotics were administered orally at a dose of 10^8^ CFU per day. Another group was treated with multiple antibiotics (MAB), and a control group was given sterile water and sterile feed without MAB treatment. It was found that probiotic bacteria treatment alleviated lung changes, reduced the concentration of IL-1β, IL-13 and IL-17 and increased IL-10 in splenocytes and bronchial lymph nodes.

### *Lactobacillus delbrueckii*

A study by Montuori-Andrade et al. [91] recently confirmed the effectiveness of the use of *Lactobacillus delbrueckii*, UFV-H2b20 strain in a mouse model of allergic airway inflammation (OVA-induced). The bacteria were administered orally. Treatment significantly reduced the lung inflammatory response: it reduced the level of IgE and the eosinophils, monocytes and alveolar macrophages levels and increased the IFN-γ/IL-4 cytokine ratio, while increasing IL-10 concentration and CD39 + CD73 + Tregs number in the lung.

### *Lactobacillus casei*

Li et al. [92] investigated the preventive effect of *Lactobacillus casei* strains on HDM-induced asthma. Probiotics were administered orally for a week before intervention. Positive controls were Ketotifen and LGG. The experimental results confirmed a reduction in the number of granulocytes and levels of Th2 and Th17 inflammatory cytokines in the lungs. Probiotics increased the secretion of immunoglobulin A (sIgA) and IL-10. Selected strains reduced the concentration of HDM-specific IgG1 and total IgE and increased the richness of intestinal microflora (mainly *Firmicutes*). The findings indicate that *L. casei3* had the greatest preventive effect by increasing the content of acetates and propionates.

Other studies [93, 94] have also confirmed the positive effects of these probiotic bacteria. Probiotics have been found to reduce oxidative stress and increase the antioxidant defense in asthmatic mice. Their use resulted in a statistically significant increase in GPx activity and a decrease in thiobarbituric acid reactive substances (TBARS).

### *Lactobacillus bulgaricus*

In 2019, Anatriello et al. [95] found that *Lactobacillus bulgaricus* N45.10 strain reduces eosinophilic inflammation and the appearance of peribronchial edema in allergic bronchial asthma. It also reduced the concentration of total and OVA-specific IgE and inhibited excessive airway mucus secretion. *L. bulgaricus* weakened the systemic allergic response and may also alleviate other accompanying diseases, such as food allergy. The results suggest that probiotic administration reduces the Th2 response in allergic mice by attenuating Toll-like receptor 4 (TLR4) expression on DCs. The BALF of mice treated with *L. bulgaricus* showed reduced secretion of IL-33 by epithelial cells, whose function is to activate lymphoid cells. The researchers propose that the anti-inflammatory actions of *Lactobacillus bulgaricus* are associated with a reduction in the transcription of *STAT-6* and *GATA-3*, associated with the exacerbation of inflammation and the allergic response. A reduction in IL-17 concentration was also observed. The study showed that this probiotic limits the participation of Th2 and Th17 cells, tipping the balance towards Th1 cells.

### *Lactobacillus reuteri*

Li et al. [96] examined the effect of administering six *Lactobacillus* species (*L. reuteri, L. rhamnosus*, *L. fermentum*, *L. casei*, *L. gasperi* and *L. salivarius*) on the course of HDM-induced asthma in mice. *L. reuteri* reduced airway inflammation, total IgE and HDM-IgG1, and pro-inflammatory cytokine concentrations. In addition, only *L. reuteri* increased butyrate production by intestinal microorganisms. This probiotic may reduce the risk of developing asthma by modulating specific gut microbiota to improve the immune environment of the lungs.

A recent Korean study [97] showed that three lactic acid bacteria species, *viz. L. rhamnosus* GCWB1156, *L. plantarum* GCWB1001 and *P. acidilactici* GCWB1085, prevent inflammation exacerbated by DEPM administration in asthmatic mice.

Recent studies have examined the potential of bacteria from the *Bifidobacteriaceae* family in the treatment of asthma. Various *Bifidobacterium* genera have been found to exert therapeutic effects, e.g. *Bifidobacterium breve* MRx0004 reduces tissue infiltration by eosinophils and neutrophils [98], and *Bifidobacterium longum* alleviates lung inflammation in mice [99]. In addition, *Bifidobacterium infantis* inhibits the secretion of IL-4, IL-13 and IgE in response to the allergen [100].

### *Bifidobacterium infantis*

Wang et al. [101] assessed the therapeutic properties of *Bifidobacterium infantis* in the course of allergic asthma. Mice receiving monelukast (Mon) and *B. infantis* demonstrated significantly lower reactivity compared to an OVA group, and the group taking Mon alone achieved similar results to controls; this indicates that probiotic can reduce AHR in a similar way to the leukotriene receptor antagonist Mon. In the Mon and *B. infantis* group, the BALF had lower levels of eosinophils, neutrophils and macrophages. The group treated with Mon and probiotic demonstrated less cellular infiltration, and a thinner layer of muscles and airway walls. The group receiving the probiotic had higher concentrations of INF-γ and IL-2. This suggests that the therapeutic action of *B. infantis* is most likely based on changes in the Th1/Th2 balance. Further studies should assess the possible drug resistance of the probiotic strains used and determine a safe dose that could be used in asthmatics.

### *Enterococcus faecalis*

In 2023, Arntz et al. [102] evaluated the impact of *Enterococcus faecalis* on SCFAs concentrations in mothers and the risk of asthma in children. A mouse model was used in which pregnant or lactating animals were orally administered a probiotic every day from day 6 of pregnancy to day 21 after birth (10^6^, 10^7^ or 10^8^ CFU). Intervention resulted in higher levels of SCFAs in the offspring feces after weaning and after allergy induction. No differences in disease severity were observed between the group receiving probiotics and controls; this may be related to the insufficient increase in SCFAs concentration, which did not reach the immuno-protective level.

### *Saccharomyces cerevisiae*

A recent study [103] examined the optimal dose and administration regimen of *Saccharomyces cerevisiae* UFMG A-905 for asthma prevention. The probiotic was administered by gavage in two treatment regimens– every day or three times a week. In the first regimen, different concentrations of probiotic (10^7^, 10^8^, or 10^9^ CFU/ml) were administered 10 days before sensitization and during the challenge. In the second regimen, the probiotic was administered for five weeks at a concentration of 10^9^ CFU/ml starting 14 days prior to the first sensitization. Importantly, in both regimens, administration of 10^9^ CFU/ml of probiotic significantly reduced bronchial AHR. Moreover, only daily treatment with the highest dose significantly reduced IL-4, IL-5 and IL-13 concentrations, as well as the total cell count and eosinophils number in the BALF. The study confirms that *S. cerevisiae* UFMG A-905 has a positive effect on preventing the development of asthma, but it is dose-dependent.

**Fig. 1:**
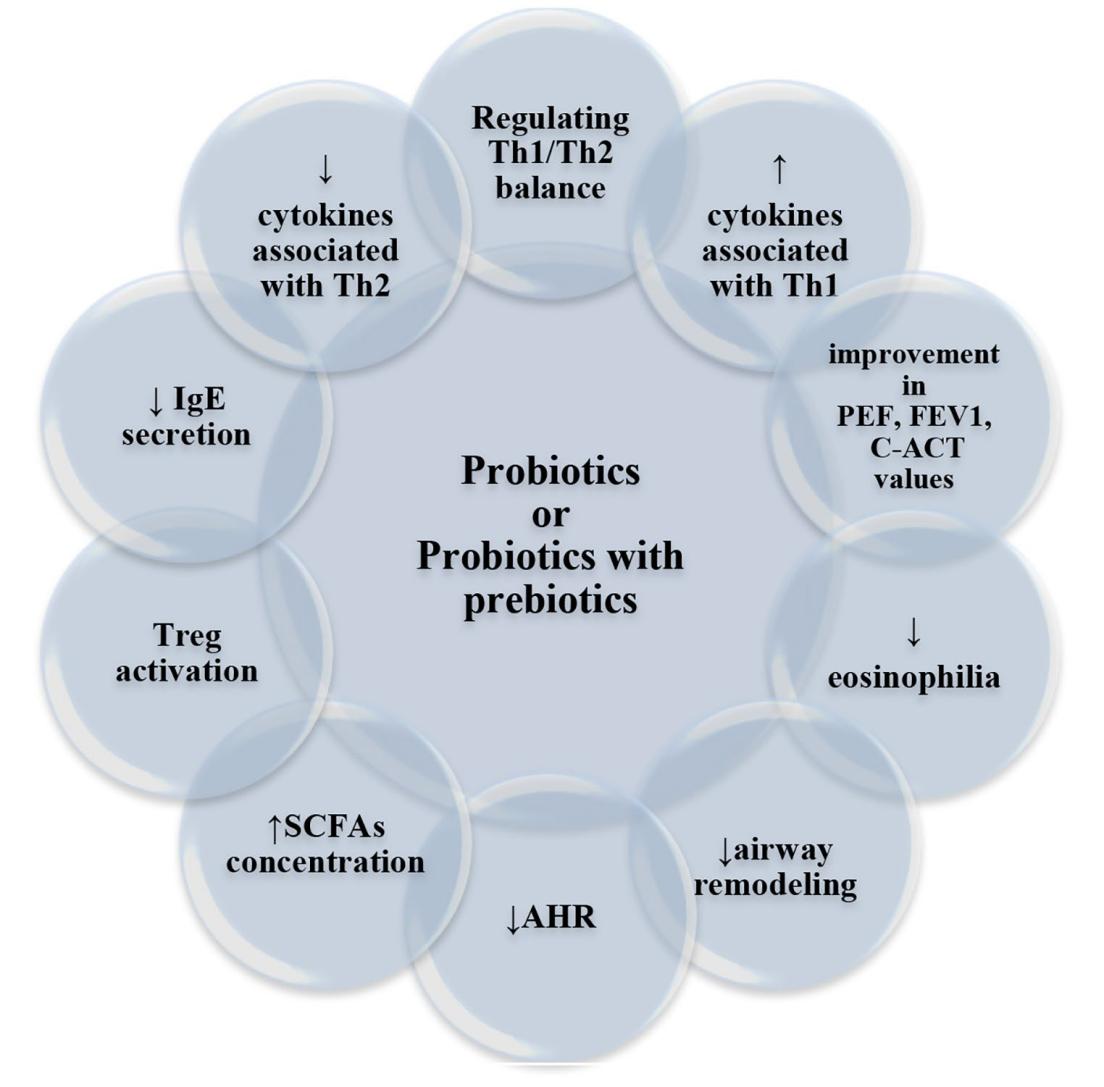
The potential mechanisms and effects of probiotics or the combination of probiotics with prebiotics in the course of asthma AHR - airway hyperresponsiveness; IgE - immunoglobulin E; SCFAs - short-chain fatty acids; Th1 - type 1 helper cells; Th2 - type 2 helper cells; Tregs - regulatory T cells; ↑- increase; ↓- decrease/ reduction

Recent studies indicate that combinations of probiotic genera, species or strains are highly effective at treating bronchial asthma [97, 104]. Moreover, better therapeutic properties occur when probiotics are combined with prebiotics that enhance their action (Fig. 1). This combination has a better effect on inhibiting Th2 cells [83]. The administration of probiotics alone significantly reduces AHR in asthmatic mice, but not the administration of prebiotics alone [31]; however, their combination diminishes pulmonary AHR after OVA sensitization and challenge in mice [105]. Vos et al. [106] proved that a specific mixture of oligosaccharides containing scGOS and lcFOS reduces lung resistance and BALF inflammatory cells in a mouse model of OVA-induced asthma. Abbring et al. [107] reported that administration of lcFOS in combination with *Bifidobaterium breve* M-16 V alleviates pulmonary resistance and airway inflammation. Another study examined the effects of combining probiotics with popular bacterial lysates [108].

In vivo studies on animal models assessing the use of probiotics in asthma are presented in Table 1.

**Table 1:** In vivo studies on animal models assessing the application of probiotics in the course of asthma; based on data from the last five years

Probiotic genus/species/strain(s)	Experimental model	Effect	References
*Lactobacillus rhamnosus* GG *Lactobacillus rhamnosus* GR-1	BALB/cOlaHsd male mice, BP aeroallergen	Beneficial (↓BALF eosinophils count, ↓lung IL-13 and IL-5, ↓AHR - only LGG)	[81]
*Lactobacillus rhamnosus* GG and/or turmeric (TP) (potential prebiotic compound)	BALB/c male mice, HDM	Beneficial (with TP- ↓AHR; ↓eosinophilia, IL-5, IL-13, CCL17)	[83]
*Lactobacillus rhamnosus* GG and/or prednisolone	BALB/c female mice, Der p 2	Beneficial (↓prednisolone dose; 50 µl of prednisolone + LGG-↓airway resistance and serum IgE, IgG1; ↓IL-4, IL-5, IL-6, IL-8, IL-13 and IL-17, ↑IgG2a in serum)	[84]
*Lactobacillus rhamnosus* 76 (LR76)	BALB/c female mice; OVA	Beneficial (alleviating inflammation; ↓ IL-4, IL-5, IL-13, IL-25 inhibition of mucus secretion in airway epithelial cells by downregulating the STAT6/SPDEF pathway)	[85]
The wild-type and recombinant *Lactobacillus rhamnosus* GR-1	BALB/cOlaHsd mice, Bet v 1	Beneficial (preventing deterioration of respiratory function and promoting the immunity of the intestinal microbiome)	[86]
*Lacticaseibacillus paracasei* K47	BALB/c mice, OVA	Beneficial (modulating host immune response to alleviate AHR and inflammation ↓serum total IgE, OVA-specific IgE and OVA-specific IgG1; regulating Th1/Th2 balance)	[87]
*Lactobacillus paracasei* 33 (LP33)	SD male rats, OVA	Beneficial (↓BALF total number of inflammatory cells, lymphocytes and eosinophils,↓IgE and cytokine levels in Th2 cells)	[88]
*Lactobacillus plantarum* CQPC11	BALB/c mice, OVA	Beneficial (↓BALF TNF-α, IL-4, IL-5, IL-6, 13; ↓histological edema, OVA-specific IgE, IgE, and IgG1)	[89]
*Lactiplantibacillus plantarum* RGU (Lp-1)	BALB/c mice, OVA	Beneficial (↑IL-10 expression, ↓IL-1β, IL-13 and IL-17 in lymphoid tissue)	[90]
*Lactobacillus delbrueckii* UFV-H2b20	BALB/c female mice, OVA	Beneficial (↓lung IgE, eosinophils, monocytes and alveolar macrophages; ↑IFN-γ/IL-4 cytokine ratio, ↑lung IL-10, CD39 + CD73 + regulatory T cells)	[91]
*Lactobacillus casei*	BALB/c mice, HDM	Beneficial (↑acetate and propionate content depending on the strain; ↑sIgA and IL-10)	[92]
*Lactobacillus bulgaricus* N45.10	BALB/c-mice, OVA	Beneficial (↑anti-inflammatory cytokines: T-bet, Foxp3; attenuating inflammation and airway remodeling by interfering with Th1/Th2 cytokines and STAT6/T-bet transcription factors)	[95]
*Lactobacillus rhamnosus* *Lactobacillus fermentum* *Lactobacillus salivarius* *Lactobacillus gasseri* *Lactobacillus reuteri Lactobacillus casei*	BALB/c female mice, HDM	Beneficial (prevention of asthma - *L. reuteri* only; ↑butyrate production, alleviation of airway inflammation and Th2 response in lung tissues)	[96]
*Lactobacillus plantarum* GCWB 1001 *Pediococcus acidilactici* GCWB1085 *Lactobacillus rhamnosus* GCWB1156	BALB/c mice, OVA, DEPM	Beneficial (↓induced inflammatory infiltration, goblet cell hyperplasia, airway remodeling, levels of pro-inflammatory cytokines and chemokines in BALF)	[97]
*Bifidobacterium infantis* CGMCC313-2	BALB/c mice, OVA	Beneficial (↓the infiltration of inflammatory cells, promoting the Th1 immune response and inhibiting the Th2 immune response)	[100]
*Bifidobacterium infantis*	BALB/c male mice, OVA	Beneficial (↓AHR, ↓ cytokines associated with Th2- ↓ BALF/lung IL-4, IL-5, IL-13; ↑cytokines associated with Th1-↑ BALF/lung IFN-γ; ↓the content of eosinophils, neutrophils and macrophages in BALF)	[101]
*Enterococcus faecalis*	BALB/c mice, OVA	Neutral (↑SCFAs concentration in offspring; no protection against allergic asthma)	[102]
*Saccharomyces cerevisiae* UFMG A-905	BALB/c male mice, OVA	Beneficial (↓AHR and lung inflammation in a dose-dependent manner)	[103]
Mixed strains: *Lactobacillus gasseri* LK001 *Lactobacillus salivarius* LK002 *Lactobacillus johnsonii* LK003 *Lactobacillus paracasei* LK004 *Lactobacillus reuteri* LK005 *Bifidobacterium animals* LK011	BALB/c male mice	Beneficial (immunomodulatory effects; probable mechanism of action - accumulation of gutprimed Foxp3 + Treg induced by MLN CD103 + DC, which can migrate to the lung through the lymph and/or bloodstream)	[104]
Mixed strains: *Lactobacillus acidophilus* LA-5 *Lactobacillus rhamnosus* GG *Bifidobacterium animalis* subspecies lactis BB-12 or FOS and GOS (prebiotics)	BALB/c male mice; OVA-LPS	Beneficial (↓AHR, ↓BALF eosinophils, ↓IL-17, ↓EPO activity - only probiotics; prebiotics -↑ BALF IFN-γ, control of the level of IL-4, IL-5, IL-13, IL-25 and IL-33, leukotrienes, AKT, NLR3, NF-κB, MyD88, MUC5a gene expression, peribronchial inflammation; ↑expression of the IL-38 gene)	[31]
*Bifidobacterium breve* Bif11 *L. plantarum* LAB31 with IMO (potential prebiotic compound)	BALB/c female mice; OVA	Beneficial (↓AHR, modulation Th2 immune response potentially through SCFAs production, suppression of OVA-induced airway inflammation)	[105]
*Bifidobacterium tetravaccine* and a mixture of bacterial lysates	BALB/c female mice	Beneficial (↑proportion of Tregs in peripheral blood; ↓risk of asthma only in offspring of mothers with a high microbiological load)	[108]

AHR - airway hyperresponsiveness; BALF - bronchoalveolar lavage fluid; FOS - fructo-oligosaccharides; GOS - galacto-oligosaccharides; HDM - house dust mites; IL - interleukin; IMO-isomaltooligosaccharide; OVA - ovalbumin; SCFAs - short-chain fatty acids; Th1 - type 1 helper cells; Th2 - type 2 helper cells; Tregs - regulatory T cells

↑- increase; ↓- decrease/ reduction

## Human trials assessing the use of probiotics in asthma

### *Lactobacillus paracasei* with *Lactobacillus fermentum*

Huang et al. [109] published the results of a randomized, placebo-controlled trial on the effect of *Lactobacillus paracasei*, *Lactobacillus fermentum* and a combination of both strains, on the severity of asthma in children, and on markers of the immune system. The study included 152 patients with sporadic or moderate asthma. The participants received probiotics for three months. The severity of the disease decreased after therapy in all probiotic groups. Additionally, in children taking both probiotics, PEF values improved significantly. Also, this group demonstrated a significant reduction in blood IgE concentration. The probiotic groups demonstrated lower stool *Clostridium* bacteria content compared to placebo, although the difference was statistically insignificant. In all groups receiving the bacteria, childhood asthma control test (C-ACT) values increased and asthma improved. The described clinical trial confirmed that both strains can reduce the severity of asthma in children, regardless of the additional drugs used, and the effects were most pronounced when given in combination. This may be due to the fact that the microbiome varies between individuals, and perhaps the use of two similar, beneficial strains increases the chance that at least one of them will adapt to the microflora of a specific patient and modulate it.

### *Lactobacillus salivarius* with *Bifidobacterium breve*

Drago et al. [110] investigated whether a probiotic mixture of *L. salivarius* LS01 (DSM 22,775) and *B. breve* B632 (DSM 24,706) could reduce asthma exacerbations in children, followed in a primary care setting. A randomized, placebo-controlled, double-blind study included 422 children receiving probiotics twice daily for eight weeks. The authors reported that probiotic supplementation is safe and significantly reduces the frequency of asthma exacerbations by more than one third.

### *Lactobacillus reuteri*

A small study [111] in adults with mild asthma showed that *L. reuteri* DSM-17,938 did not affect airway nerves, smooth muscle, sputum inflammatory cells, skin, or T cells responses. The study included 15 patients. The authors used a randomized, double-blind, placebo-controlled, two-way cross-over study of patients with mild allergic asthma, consisting of four visits; the aim was to compare dose cough responses with inhaled capsaicin after one month of probiotic treatment vs. placebo. The beneficial results of preclinical studies in this area have not been confirmed.

Another study [112] evaluated the efficacy of *L. reuteri* DS 17,938 as an adjunctive therapy in the treatment of asthmatic children and adolescents. A pilot longitudinal, experimental and nonrandomized study included 30 patients aged 6 to 17 years. Asthmatics were examined after at least 60 days. The authors observed an increase in asthma control test scores and a decrease in the number of symptoms in the probiotic supplementation group. An increase in PEF was also observed in this group.

### *Lactobacillus rhamnosus*

A randomized, double-blind controlled trial [113] was conducted to determine whether the administration of probiotics in the first 6 months of life reduces the risk of developing asthma, eczema and rhinitis in children. The study included 184 children receiving LGG and inulin daily for the first six months of life. For high-risk infants, early administration of probiotic supplements did not prevent the development of asthma at two years of age.

### *Lactobacillus rhamnosus* with *Bifidobacterium lactis*

In a follow-up study published in 2024 [114], children of mothers supplemented with *L. rhamnosus* GG + *B. lactis* BB12 during pregnancy did not demonstrate any improvement in the incidence of asthma. The study included 107 women who completed a questionnaire.

However, since 2015, the World Allergy Organization (WAO) has recommended the use of probiotics in pregnant women at high risk of having an allergic infant [115].

### Multi-strain probiotics

A study by Liu et al. [76] confirmed that the combination of several probiotics with budesonide alleviates asthma symptoms by modulating the gut microbiome and serum metabolome. A three-month randomized, double-blind, placebo-controlled human trial was conducted in 31 asthmatics (55 initially). Probiotic co-administration significantly decreased the fractional exhaled nitric oxide level after a month of use and enhanced the asthma control test score at the end of the intervention. The concentration of alveolar nitric oxide decreased significantly after a month of using the preparations. Symptom relief was most evident after the experiment was completed, i.e. on day 90.

In 2023, Abbasi-Dokht et al. [116] published the results of a randomized, double-blind, placebo-controlled trial. The study included 40 patients with asthma. Patients received one capsule of multi-strain probiotic (*L. casei*, *L. acidophilus*, *L. rhamnosus*, *L. bulgaricus*, *B. breve*, *B. longum*, *S. thermophilus*) and fructooligosaccharides for 8 weeks. The authors reported a significant increase in FoxP3 expression and CD4 + CD25 + FoxP3 + Tregs population and a decrease in RORγt and GATA3 expression. Furthermore, probiotic supplementation significantly improved forced expiratory volume (FEV) and forced vital capacity (FVC).

Hassanzad et al. [117] analyzed the efficacy and safety of a probiotic consisting of *L. casei*, *L. acidophilus*, *L. rhamnosus*, *L. bulgaricus*, *B. infantis*, *B. breve*, *S. thermophiles* and fructooligosaccharide in the treatment of asthmatic children. A six-month double-blinded, randomized, placebo-controlled clinical trial was performed on 100 children aged 12 years and younger with mild to moderate asthma. The data showed that synbiotics significantly reduce the number of outpatient visits to the hospital for asthma-related problems, while rarely causing any side effects.

McLoughlin et al. [118] investigated the effects of inulin supplementation, with or without probiotic, on plasma SCFAs, airway inflammation, asthma control and gut microbiome in asthmatics adults. In the randomized, double-blinded, placebo controlled three-way cross-over trial *L. acidophilus* LA-5, *L. rhamnosus* GG, *B. animalis* subspecies *lactis* BB-12 and/or inulin were used. The study involved 17 patients with stable asthma who received probiotics three times a day for seven days. There was no difference in change in total plasma levels of SCFAs in the placebo *vs.* inulin + probiotic group. The authors described an improvement in airway inflammation, asthma control and gut microbiome composition after inulin administration.

### The effectiveness of bronchial asthma therapy using probiotics

A meta-analysis by Lin et al. [119] examined the effectiveness of bronchial asthma therapy using probiotics in children. For inclusion, all trials had to be randomized and controlled, the participants had to be aged under 18 years and diagnosed with asthma, and probiotics had to be the only form of intervention in the trial. Eleven studies meeting these criteria were analyzed. The research included 910 children. The extremely high heterogeneity was noted for most parameters, which may account for differences in effect estimates. The analysis yielded ambivalent results: although the results suggest the use of probiotics appears to have a positive effect on reducing the number of asthmatic episodes in children, the method of assessment and its uniformity between studies remain unclear, insofar that probiotics did not appear to have any influence on C-ACT parameters or the presence of asthma symptoms.

Due to the small number of studies, no intergroup analysis was possible, and the results may have been influenced by the clinical diversity of patients, the choice of a specific probiotic strain or the duration of the intervention. Two studies showed an improvement in FEV1 and PEF parameters after using a probiotic. Treatment yielded positive effects in patients with mild asthma, which suggests that the results may depend on the level of advancement of the pathology. It was also found that age and sex may be important. Moreover, probiotics significantly reduce the concentration of IL-4. However, significant heterogeneity was observed between studies due to the use of different strains of probiotics [119].

A 2021 study by Wawryk-Gawda et al. [120] indicates that administering probiotics in the first months after birth does not reduce the risk of developing asthma in the first years of life in high-risk children.

A more recent meta-analysis based on asthmatics of all ages found that probiotic strains, administered mainly orally, reduce inflammation, asthma symptoms and the number of asthma attacks but do not affect lung function [121].

**Fig. 2:**
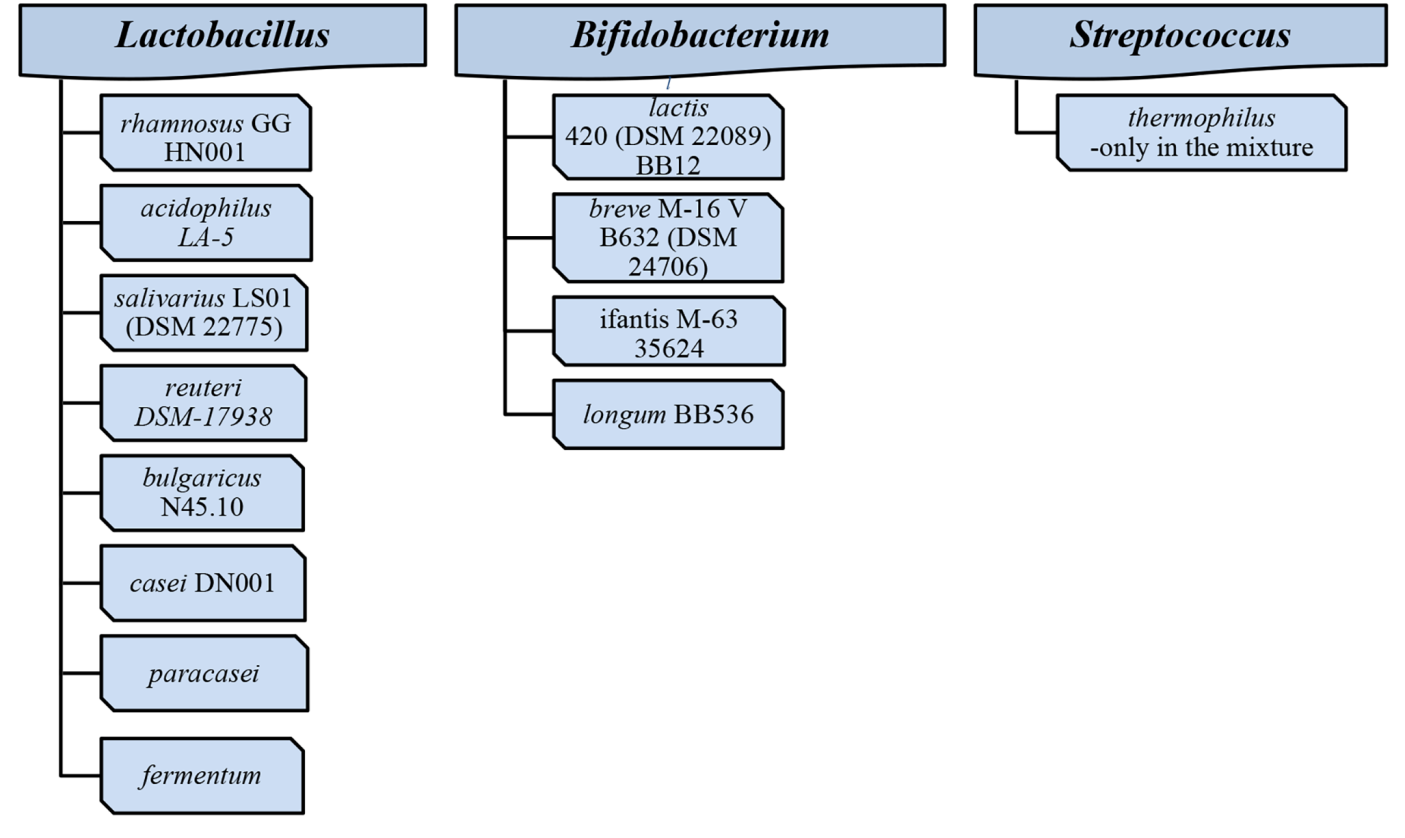
The most frequently-used probiotic genus/species/strains in asthma (human trials); based on data published in recent years

The most frequently-used probiotics in clinical trials in asthma are presented in Fig. 2.

## Conclusions

The dynamic growth of interest in probiotics and their therapeutic potential in asthma is best illustrated by the growing amount of scientific publications on this topic. Studies examined the traditional oral administration and more innovative intranasal or even intratracheal administration of probiotic microorganisms. Most published results are based on animal models of allergic asthma. It has been demonstrated that selected probiotic bacteria exhibit anti-inflammatory activity and may have a beneficial effect on AHR. Probiotics reduce the concentration of IgE in the blood, limit the infiltration of eosinophils in the airway and restore Th1/Th2 cellular balance. In addition, the administration of *L. bulgaricus* or *L. rhamnosus* results in a reduction in airway mucus secretion, which may translate into improved patient well-being in clinical practice. Intranasal application of bacteria may contribute to a more effective stimulation of the processes taking place in the lymph nodes, but it may result in a potential excessive distribution of bacteria in the body.

Although clinical studies involving the use of probiotics in asthma are sparse, several randomized, double-blinded and placebo-controlled have been published recently. The results confirm that probiotic supplementation is safe and significantly reduces the frequency of asthma exacerbations. A relatively small number of studies indicate that the use of probiotics can improve PEF, FEV1 and C-ACT values and reduce IL-4 and blood IgE levels. There are discrepancies regarding the effectiveness of probiotics in asthma preventing.

However, our current understanding of the individual response to probiotic therapy, and the effects of its combination or doses, remains insufficient, and few clinical trials are available to draw clear conclusions. Hence, more research is needed to confirm the beneficial effects of microorganisms for them to be regarded as therapeutic options.

### Future perspectives

Animal studies indicate that the application of probiotics could be considered a supportive treatment of asthma in the future. However, comparable clinical trials are still relatively few and inconclusive. Although the results of recent human studies are encouraging, further standardization is needed, especially with regard to the therapy regimen and the choice of strains for specific patient groups. Also, the effectiveness and safety of long-term use of probiotics in specific conditions need to be determined.

## Data Availability

Not applicable.

## Abbreviations

ACT: Asthma control test
AHR: Airway hyperresponsiveness
BALF: Bronchoalveolar lavage fluid
C-ACT: Childhood asthma control test
DCs: Dendritic cells
FEV: Forced expiratory volume
FOS: Fructo-oligosaccharides
FVC: Forced vital capacity
GOS: Galacto-oligosaccharides
IgA: Immunoglobulin A
IgE: Immunoglobulin E
IL: Interleukin
INF-γ: Interferon gamma
IL: Interleukin
NK: Natural killer
OVA: Ovalbumin
PEF: Peak expiratory flow rate
SCFAs: Short-chain fatty acids
Th1: Type 1 helper cells
Th2: Type 2 helper cells
Th17: T helper type 17
TNF-α: Tumor necrosis factor-alpha
Tregs: Regulatory T cells
